# Minimum information for reporting on the TEER (trans-epithelial/endothelial electrical resistance) assay (MIRTA)

**DOI:** 10.1007/s00204-024-03879-z

**Published:** 2024-10-04

**Authors:** Monita Sharma, Erin Huber, Emma Arnesdotter, Holger P. Behrsing, Adam Bettmann, David Brandwein, Samuel Constant, Rahul Date, Abhay Deshpande, Eric Fabian, Amit Gupta, Robert Gutierrez, Arno C. Gutleb, Marie M. Hargrove, Michael Hollings, Victoria Hutter, Annie M. Jarabek, Yulia Kaluzhny, Robert Landsiedel, Lawrence Milchak, Robert A. Moyer, Jessica R. Murray, Kathryn Page, Manish Patel, Stephanie N. Pearson, Elijah J. Petersen, Emily Reinke, Nuria Roldan, Clive Roper, Jamie B. Scaglione, Raja S. Settivari, Andreas O. Stucki, Sandra Verstraelen, Joanne L. Wallace, Shaun McCullough, Amy J. Clippinger

**Affiliations:** 1 PETA Science Consortium International e.V., 70499 Stuttgart, Germany; 2 Exposure and Protection, RTI International, 3040 East Cornwallis Road, Durham, NC USA; 3 Environmental Research and Innovation (ERIN) Department, Luxemburg Institute of Science and Technology, 5 Avenue Des Hauts-Fourneaux, 4362 Esch-Sur-Alzette, Grand Duchy of Luxembourg; 4 Institute for In Vitro Sciences, Inc., Gaithersburg, MD 20878 USA; 5 3M Company, St. Paul, MN 55144 USA; 6 Epithelix Sàrl, Chemin Des Aulx 18, 1228 Plan-Les-Ouates, Switzerland; 7 Jai Research Foundation, N. H. 48, Near Daman-Ganga Bridge, Valvada, Gujarat 396105 India; 8 BASF SE, Experimental Toxicology and Ecology, 67056 Ludwigshafen, Germany; 9 Life Science Research, Battelle Memorial Institute, Columbus, OH 43201 USA; 10 Materials Measurement Laboratory, National Institute of Standards and Technology (NIST), Gaithersburg, MD 20899 USA; 11 Syngenta Crop Protection, 410 Swing Rd, Greensboro, NC 27409 USA; 12 Labcorp Early Development Laboratories Ltd., North Yorkshire, HG3 1PY UK; 13 ImmuONE Ltd, Sycamore House, 16 Leyden Road, Stevenage, Herts SG1 2BP UK; 14 Centre for Topical Drug Delivery and Toxicology, University of Hertfordshire, College Lane Campus, Hatfield, Herts AL10 9AB UK; 15 Center for Public Health and Environmental Assessment (CPHEA), Office of Research and Development, U.S. Environmental Protection Agency (EPA), Research Triangle Park, Washington, NC 27711 USA; 16 MatTek Life Sciences, Ashland, MA 01721 USA; 17 Pharmacy, Pharmacology and Toxicology, Free University of Berlin, Berlin, Germany; 18 The Clorox Company, 4900 Johnson Dr, Pleasanton, CA 94588 USA; 19 Inotiv, Morrisville, NC 27560 USA; 20 Roper Toxicology Consulting Limited, Edinburgh, EH3 6AD UK; 21 ScitoVation, LLC, Durham, NC 27709 USA; 22 Corteva Agriscience, Haskell R&D Center, Newark, DE USA; 23 Environmental Intelligence Unit, Flemish Institute for Technological Research (VITO), 2400 Mol, Belgium; 24 Charles River Laboratories Edinburgh Ltd, Elphinstone Research Centre, Tranent, East Lothian EH33 2NE UK; 25 InVitroTox Solutions Consulting, Newton, USA; 26 Kimberly-Clark Corporation, Irving, USA

**Keywords:** TEER, Barrier integrity, In vitro, Respiratory toxicity, Inhalation

## Abstract

Standard information reporting helps to ensure that assay conditions and data are consistently reported and to facilitate inter-laboratory comparisons. Here, we present recommendations on minimum information for reporting on the TEER (trans-epithelial/endothelial electrical resistance) assay (MIRTA). The TEER assay is extensively used to evaluate the health of an epithelial/endothelial cell culture model and as an indicator of the potential toxicity of a test substance. This publication is the result of an international collaboration─called the RespTox (Respiratory Toxicity) Collaborative─through which twelve laboratories shared their protocols for assessing the barrier function of respiratory epithelial cells using the TEER assay following exposure to substances. The protocols from each laboratory were reviewed to identify general steps for performing the TEER assay, interlaboratory differences between steps, the rationale for differences, whether these differences impact results or cross-laboratory comparisons between TEER measurements. While the MIRTA recommendations are focused on respiratory epithelial cell systems, these recommendations can be adapted for other cell systems that form barriers. The use of these recommendations will support data transparency and reproducibility, reduce challenges in data interpretation, enable cross-laboratory comparisons, help assess study quality, and facilitate the incorporation of the TEER assay into national and international testing guidance.

**Supplementary Information:**

The online version contains supplementary material available at 10.1007/s00204-024-03879-z.

## Introduction

**Fig. 1:**
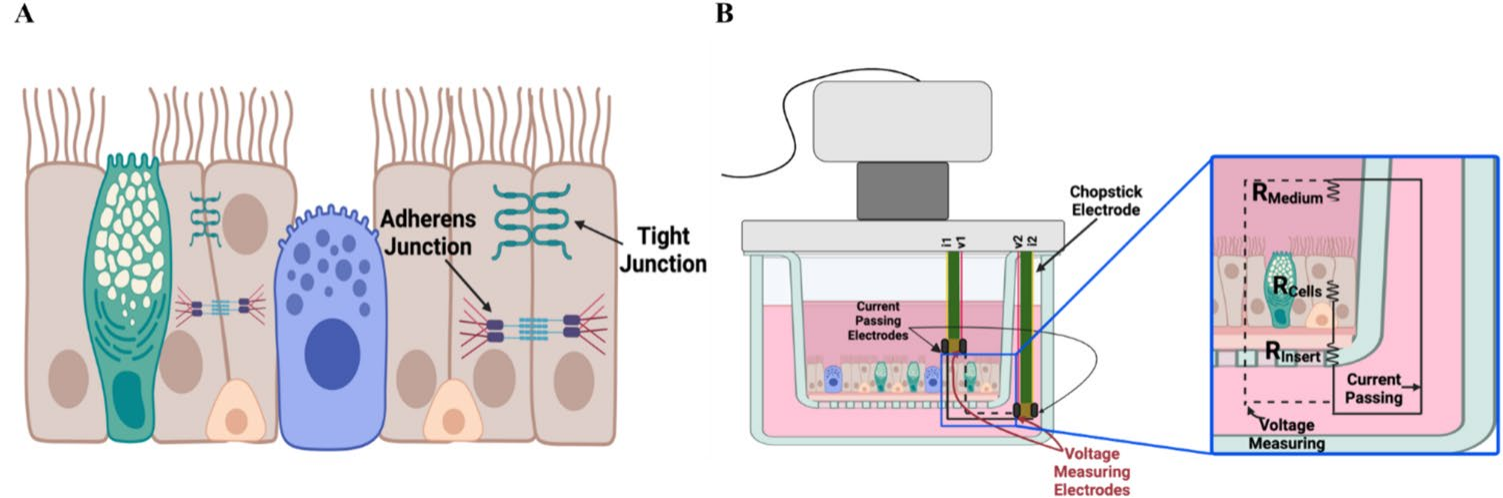
**A** Figure depicting epithelial cell barriers with tight and adherens junctions. **B** Figure demonstrating the principle of TEER measurement to quantify the electrical resistance introduced by a confluent cell layer to current passing across the apical and basolateral compartments of a porous membrane in an insert-based cell culture system. Figure was created on BioRender.com

The trans-epithelial/endothelial electrical resistance (TEER) assay is extensively used to evaluate the health of an epithelial/endothelial cell culture model and as an indicator of the potential toxicity of a test substance (Movia et al. 2020). These cells form selectively permeable, polarized barriers that separate the luminal (apical) and abluminal (basolateral) compartments throughout the body, such as the respiratory and gastrointestinal tracts, skin, brain, vasculature, and renal system. The integrity of these barrier tissues is dependent on interactions between adjacent cells (e.g., through maintenance of tight and adherens junctions) (Fig. 1A). For example, in proximal and distal airways of the respiratory tract, these barriers function to separate inhaled air (as well as any accompanying environmental materials) from the submucosal tissue and from the bloodstream at the alveoli, while also regulating the paracellular transport of ions and macromolecules (Hollenhorst et al. 2011; Folkesson and Matthay 2006). Respiratory barrier dysfunction occurs in a wide range of respiratory diseases and can result from exposure to a broad range of inhaled chemicals, particulates, and pathogens (Radbel et al. 2020; Shin et al. 2017). Evaluating the ability of a test substance to reduce barrier integrity in the respiratory tract is, therefore, of importance in both regulatory toxicology and biomedical research.

TEER is a measure of electrical resistance across one or more cell layers grown on a porous membrane after they reach confluence. The electrical resistance, which represents the impedance to current flow within a circuit, provides a quantitative measure to assess the integrity of a monolayer by evaluating its ionic conductance. A small electrical current is passed between two electrodes that are positioned at opposing sides of the barrier (apical and basolateral), and voltage is measured (Fig. 1B). Using Ohm’s law (represented by the equation: V = IR, where V is voltage, I is current, and R is resistance), TEER output is computed in Ohm·cm^2^. A higher TEER value indicates a tight cellular barrier. TEER assay output is an indicator of barrier integrity. In some studies, changes in barrier integrity measured in vitro are qualitatively comparable to indicators of impaired barrier integrity in in vivo animal and human studies such as interstitial-to-luminal translocation of macromolecules (e.g., albumin in bronchoalveolar lavage fluid (BALF) following respiratory tract injury). For example, data for exposure to ozone (O_3_; an extensively studied inhaled toxicant) show reduced respiratory barrier integrity in vivo in humans as evidenced by increased protein, albumin, and fibronectin levels in BALF (Kehrl et al. 1987; Koren et al. 2012; Van Bree et al. 2002; Albright et al. 2022; Que et al. 2011). Similarly, decreased TEER assay values were observed after ozone exposure in primary human bronchial epithelial cells in vitro (Bowers et al. 2021). In addition to its use in the evaluation of the effects of exposure to a test substance, TEER is also a valuable quality control measure to ensure the suitability of in vitro tissues representing biological barriers (e.g., for respiratory, oral, ocular, and dermal model systems), prior to their use in experimental studies (Guth et al. 2015; OECD 2021a, b).

While the TEER assay is widely used across scientific sectors, there are inter-laboratory variations in equipment, cell models, assay protocols, user proficiency, response characterization, and data reporting, which can limit the comparability of TEER assay data (Braakhuis et al. 2023). For example, there are different manufacturers of voltohmmeters, different types of electrodes, and even automated/high-throughput systems to measure TEER. The MIRTA recommendations focus on two specific types of electrodes but can be adapted (if needed) and applied to other TEER apparatus (Wilkinson et al. 2016; Petersen et al. 2021; RIVER 2023; OECD 2018). While some protocol variation across laboratories is expected, establishing minimum reporting recommendations—in line with those described for the comet assay (Møller et al. 2020) and microarray experiment (Brazma et al. 2001)—will further facilitate the translational value of TEER assay data. Thus, the Respiratory Toxicity (RespTox) Collaborative—an international, cross-sector consortium of experts conducting in vitro inhalation toxicity testing—was tasked with defining the minimal information that needs to be reported following TEER measurement to maximize the utility of data. All experts who contributed the protocols for and reviewed this manuscript have/are actively using TEER assay in their work (Paudel et al. 2023; McGee Hargrove et al. 2021; Sharma et al. 2023; Wallace et al. 2023; Jackson et al. 2018; Mccullough 2020; Singh et al. 2021; Petersen et al. 2021). In addition to these reporting recommendations for the TEER assay, it would be similarly useful to develop recommendations for other in vitro assays that are widely used in the inhalation toxicity field (and beyond). Streamlining how information about an assay is reported supports proper characterization of the assay and aids data transparency and reproducibility, reduces challenges in data interpretation, enables cross-laboratory comparisons, and helps assess study quality.

## Method

**Fig. 2:**
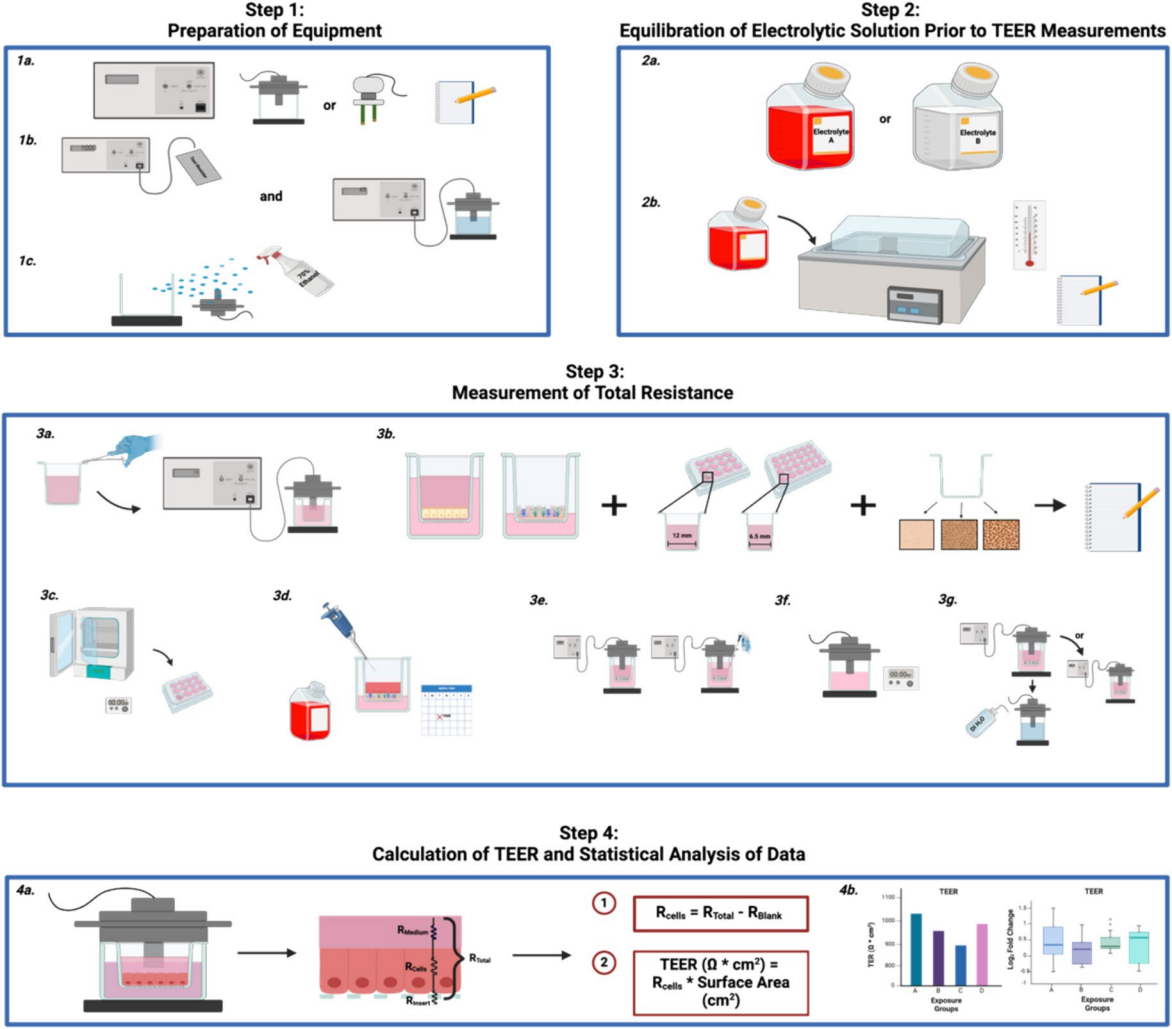
Steps involved in the measurement of total trans-barrier electrical resistance (TEER) of the biological test system. The figure was created on BioRender.com

Twelve laboratories from the RespTox Collaborative─3 M, BASF, Charles River Laboratories, Epithelix Sárl, the Institute for In Vitro Sciences, MatTek Life Sciences, the National Institute of Standards and Technology (NIST), ScitoVation, RTI International, the U.S. Environmental Protection Agency (EPA), Unilever, and the Flemish Institute for Technological Research (VITO)─shared their TEER assay protocols used for differentiated respiratory cells grown at the air–liquid interface (ALI). The protocols were compared, and several general steps were identified as common to all: (1) preparation of equipment, (2) equilibration of electrolyte solution prior to TEER measurements, (3) measurement of total resistance, and (4) calculation of TEER and statistical analysis of data. The minimal information that should be reported for each general step and justification for why it should be reported, including potential impact on results and inferences (Table 1 and Fig. 2), was generated. A template is included (see Supplementary information) as an example of how the TEER assay and data should be reported.

**Table 1:** Minimum reporting information on the TEER assay (MIRTA) (see supplementary information for an example template)

Step	General steps for measuring TEER	Suggested details to report in a publication	Rationale
Step 1	Preparation of equipment		
1a	Type of equipment	Report the manufacturer and type of voltohmmeter, type of electrodes (e.g., chamber or chopstick and catalog number), and adapter (if used)	The steps followed to perform TEER assay and the output can differ based on the type of voltohmmeter and electrodes used. Resistance measurements used to calculate TEER vary across electrode types
1b	Calibration of voltohmmeter and electrodes	Report that the voltohmmeter was calibrated (e.g., by using a test resistor) and the range of resistance used for calibration. Report the type of electrolyte solution (e.g., potassium chloride), and if available, its temperature, pH, and salt concentration used to check the electrodes before use, and whether a calibration insert was used. Maintain a historical record of calibration readings and blank readings	Calibration ensures the measurements are accurate and consistent with previous measurements. Electrode function can be checked using electrolyte solutions with varying salt concentrations or pH and the measured values differ based on the solutions used. Historical record will ensure capturing changes in equipment
1c	Cleaning and disinfection of electrodes	Report the cleaning and disinfection procedures for the electrodes	Several different protocols and solutions can be used to clean and disinfect the electrodes, some of which may corrode the electrodes over time, leading to drift in the measurements
Step 2	Equilibration of electrolyte solution prior to TEER measurements		
2a	Type of electrolyte solution	Report the type and concentration of electrolyte solution (s) used	The type of electrolyte solution (s) used in the TEER assay has been shown to impact the resistance
2b	Temperature of electrolyte solution	Report the temperature of electrolyte solution (s) used	The temperature of electrolyte solution (s) used in the TEER assay has been shown to impact the resistance
Step 3	Measurement of total resistance		
3a	Measurement of blank resistance	Report the blank measurement (see Steps 3e and 3f to measure blank resistance)	The blank resistance is required to calculate resistance and can differ based on the properties of the membrane
3b	Measurement of total resistance of the biological test system	Report the biological test system, specifications of inserts (including manufacturer, size, surface area, and membrane type (g, material, thickness, pore size, and pore density), whether the inserts are coated prior to use, and cell culture reagents (e.g., medium and supplements) used. For commercially available systems, report the reference TEER value (if provided by the manufacturer and whether the reference TEER value was experimentally confirmed) and, when available to the user, report the specifications of inserts. Documentation of negative and positive controls including historical data	TEER assay measurements can vary between different biological test systems as can the blank resistance measurements based on the type/format of permeable inserts used. Documentation of negative and positive controls as well as historical values will help translation of studies
3c	Placement of the biological test system out of the incubator	Report the room temperature and total duration for which the test system was outside the incubator in preparation for TEER measurements, including the time between the first and last measurement	Time outside of the incubator may alter TEER assay measurements due to changes in the temperature and pH of the test system
3d	Rinsing the test system	Report details of how and when the biological test system was washed and for how long before the measurement	TEER assay measurement may differ based on if, and when, the biological test system is apically washed or when the medium is changed before measurement
3e	Apical and basolateral compartments during measurement	Report the type and volume of electrolyte solution added to both the apical and basolateral compartments of the test system for TEER measurements	TEER assay measurements can vary based on the type and volume of the electrolyte solution used
3f	Equilibration of electrodes and conduct measurement	Report if and how the electrodes were equilibrated. Report the solution used for equilibration	The duration of the equilibration of the electrodes in the electrolyte solution may impact the measurements
3 g	Treatment of electrodes between measurements	Report whether the electrodes are rinsed and dried, equilibrated, or otherwise treated between measurements. If so, report the solution used to rinse electrodes between measurements	If electrodes are rinsed between measurements, the solution used for rinsing should be reported, as it may alter subsequent measurements
Step 4	Calculation of TEER and statistical analysis of data		
4a	Calculation of TEER	Report the equation used	The equation shows the details of how TEER assay values are derived
4b	Statistical analysis of results	Report the statistical analysis applied	To enable data comparison across studies, a description of the statistical model used with goodness of fit, as well as its justification, along with raw values (with the number of technical and biological replicates) must be reported

## Recommendations on minimum information for reporting on the TEER assay (MIRTA)

### Preparation of equipment

The first general step in the TEER assay is preparation of the equipment. TEER can be measured using different apparatuses, ranging from various types of commercially available equipment to customized options. Although there are some high throughput/automated options available, the more commonly used set-up includes a voltohmmeter and electrodes (chopstick electrodes or chamber electrodes; Fig. 1) (Mccullough 2020). TEER assay measurements vary depending on the type of electrode (Sheller et al., 2017), thus, the type of equipment used for the TEER assay must be reported (Step 1a).

**Fig. 3:**
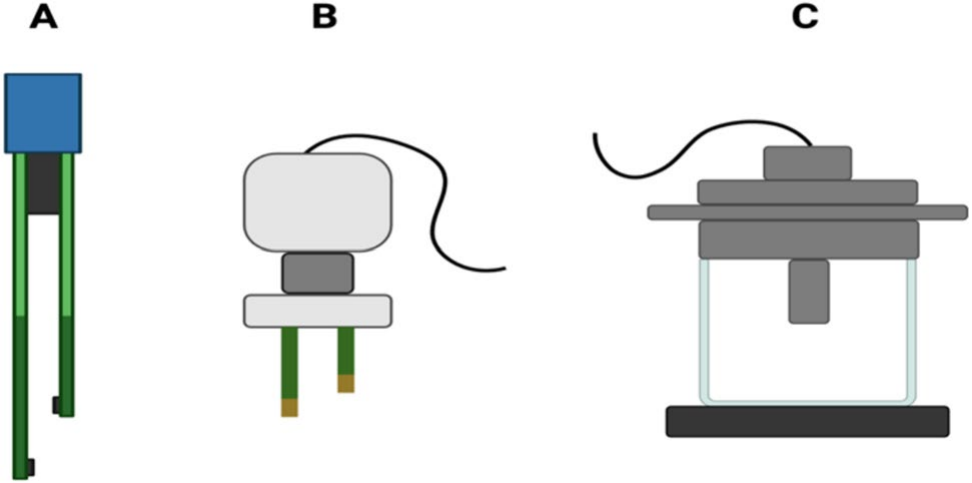
Different types of electrodes used for TEER. **A**–**B** are chopstick electrodes and **C** depicts a chamber electrode. Figure was created on BioRender.com

Before conducting the TEER assay, the voltohmmeter and electrodes should be calibrated to ensure the accuracy of instrumentation and consistency of observations across experiments (Step 1b). Calibration of the voltohmmeter is conducted by measuring the resistance of at least one test resistor and then adjusting the voltohmmeter to the calibrated value. Electrode functioning can be calibrated or checked using standard electrolyte solutions with varying conductance. Conductance depends on the types of salts in the solution (e.g., potassium chloride), the concentration, the pH, and the temperature of the solution. Certain voltohmmeters (e.g., Millipore ERS 3.0) are able to record the temperature during measurements. Regardless of the apparatus used, a calibrated system (voltohmmeter and electrodes) from any manufacturer should report electrical resistance within an acceptable range when used by proficient laboratory personnel (e.g., based on historical data and/or range provided by the manufacturer), as long as other factors (e.g., the solutions historically used for calibration) are kept constant. Of note here is that keeping a historical record of calibration readings (for internal use) can confirm that the equipment is functioning as expected. Further, reporting of values for negative and positive controls can help characterize the system and aid establishment of benchmark response levels critical for translation in risk assessment. In addition to the equipment type, it should be reported that the apparatus was calibrated, including the details related to the test resistor(s), the type of electrolyte solutions used to calibrate the electrodes, what calibration chamber, if any, was used, and frequency of calibration (e.g., every time the measurement is performed or after a set duration of use) (Step 1b) (Fig. 3).

Additionally, as the electrodes come in direct contact with cell culture media, they should be disinfected and rinsed before use to prevent contamination of cultures (e.g., by dipping them in 70% *v*/*v* ethanol or isopropyl alcohol, 5% *w*/*v* sodium hypochlorite, or 0.55% *w*/*v* ortho-phthalaldehyde), if the measurements are not at the terminal time of the assay. Since there are several types of disinfectants that may be used, and some of which can corrode (e.g., alcohol-based disinfectants) the electrodes, the method and solutions used to disinfect and clean for each study should be reported (Step 1c).

## Equilibration of electrolyte solution prior to TEER measurements

Several different electrolyte solutions/buffers can be used for a TEER assay. Examples include cell culture media, Earle's Balanced Salt Solution (EBSS), phosphate-buffered saline (PBS), and Hanks' Balanced Salt Solution (HBSS). The type (i.e., composition) of the electrolyte solution used impacts the TEER assay measurements and, therefore, must be reported (Step 2a). Ideally, electrolyte solutions used for a TEER assay should be of similar osmolarity and pH as the cell cultures to prevent potentially impacting the barrier function, causing abrupt fluctuations in measurements, and thereby confounding the interpretation of study data. Further, the electrolyte solution used for the TEER assay should contain calcium and magnesium to avoid destabilizing cell–cell and cell-substrate interactions.

Additionally, the electrolyte solution is either warmed to 37 °C or room temperature before use for TEER measurements. Since it has been shown to affect TEER assay values (Blume et al. 2010), the temperature of the buffer at the time of the measurement should be reported (Step 2b). Certain voltohmmeters (e.g., Millipore ERS 3.0) can record the temperature of the solution simultaneously and therefore capture a potential change in temperature between the start and end of the TEER assay. Certain sterile benches may include heated panels, or one can use a hot plate set to 37 °C to avoid the cell culture medium from cooling. If such equipment is used, it should be reported.

## Measurement of total resistance

Before measuring the total resistance of the biological test system, blank resistance is measured. Blank resistance is the resistance of a semipermeable membrane insert without cells, and it is subtracted from the total resistance measurement with cells to determine the final TEER value. Blank measurements vary based on the type of membrane insert used (e.g., pore diameter, density, membrane material, coating applied to the membrane, and the insert diameter) (Vigh et al. 2021; Karakocak et al. 2023) as well as the type and volume of electrolyte solution used. Therefore, blank resistance should be reported every time the instrument is used (Step 3a) before measuring the resistance of the biological test system. Keeping an internal record of blank readings can confirm that the readings are within the expected range.

The TEER assay can be used with various types of cell-based biological systems, including those that are commercially available and those that are developed in-house (Wiese-Rischke et al., 2021). TEER assay measurement of a biological test system involves several steps, including transporting the test system from the incubator to a biological safety cabinet or bench top, rinsing the test system apically and basolaterally with appropriate solution (see Step 2), adding the electrolyte solution(s) to the apical and basolateral sides, performing the measurement, replacing the solution with fresh culture medium (if applicable), and putting the test system back in the incubator. The measured TEER assay values can differ between biological test systems based on the cell types, properties of cell culture inserts (material type, coating, dimensions, pore size, and pore density) on which they are cultured, and reagents (e.g., medium and supplements) used (Barosova et al. 2021; van der Valk et al. 2018; Leung et al. 2020; Vigh et al. 2021; Karakocak et al. 2023; Srinivasan et al. 2015; Meindl et al. 2023). Therefore, the type of biological system, the cell types, and the membrane properties must be reported (Step 3b). Maintaining an internal record of TEER values for the biological system, including reporting of negative and positive controls to characterize the system being used can help confirm that the readings are within the acceptable range and aid translation of studies for risk assessment. When using commercially available test systems, the manufacturers usually provide a reference TEER value they use for quality controls. This value should be reported.

The room temperature and duration for which the test systems remain outside the incubator during the assay should be reported as it could impact the TEER assay output due to changes in the temperature (Blume et al. 2010) and pH of the test system (Step 3c and Step 2a). If devices are used (e.g., heated panels or a hot plate) to keep the temperature of the biological test system constant, those should be measured and reported. The rinsing regimen and the type of rinsing solution used also affect the TEER assay output; therefore, the details of when the test system is rinsed, and the solution used must be reported (Step 3d). Furthermore, the volume of electrolyte solution added to the well apically and basolaterally depends on the size of the insert and must be reported (Step 3e). Before performing the measurement, the electrodes are equilibrated according to the manufacturer’s instructions. The solution used for equilibration should be reported. The duration for which the electrodes are in the solution and the time it takes to stabilize the reading may impact the subsequent measurements in the test system and should, therefore, be reported (Step 3f). Also, while measuring TEER, sometimes the electrodes may be rinsed, dried, disinfected, equilibrated, or otherwise treated between each well, between plates, or between treatment groups. Therefore, the regimen of how electrodes are treated between measurements and the composition of the rinsing solution when rinsed should be reported, as this may impact the final readings (Step 3 g).

## Calculation of TEER and statistical analysis of data

Calculations of the TEER assay output follow Ohm’s law such that the resistance can be calculated based on the current and voltage passing through the cell layer. Total resistance (R_total_) measures the resistance of the biological system along with resistance of the electrolyte solution (R_solution_) and the semipermeable membrane (R_membrane_) of the cell culture insert (Fig. 1B). The resistance measured from the blank insert (R_blank_) measures R_solution_ and R_membrane_. Therefore, to determine the resistance of the cells (R_cells_), R_blank_ is subtracted from R_total_ (Step 4a)(Karakocak et al. 2023):

$$R_{{{\text{cells}}}} = R_{{{\text{total}}}} {-}R_{{{\text{blank}}}}$$

TEER is then calculated by multiplying R_cells_ by the cell culture insert surface area (SA).

$$TEER \, (\Omega \cdot cm^{2} ) = R_{{{\text{cells}}}} \left( \Omega \right) \; \times \;SA\left( {cm^{2} } \right)$$

Different statistical models can be applied to analyze TEER data. For comparison across studies, the details of statistical analysis (including the number of technical and biological replicates and statistical model used) and raw TEER values must be reported (Step 4b).

## Discussion and conclusion

As a potentially non-invasive, rapid, and straightforward assay, TEER is commonly conducted and routinely measured across different time points using the same tissue (i.e., longitudinal studies). The TEER assay has been used extensively as an indicator of toxicity following exposure to a test substance and as a quality control to ensure the suitability of tissues used in in vitro studies (Guth et al. 2015; OECD 2021a, b). For example, several Organisation for Economic Co-operation and Development (OECD) test guidelines (TGs) for in vitro skin or eye testing require assessment of barrier integrity as a measure of quality and health of the test system before use (OECD 2021a; 2019; 2021b). Therefore, it is important to have minimum requirements for reporting on the study design and results from this assay. This publication outlines key steps common to TEER testing in twelve laboratories assessing the barrier function of respiratory cells. A template is provided in the Supplemental Information to facilitate the reporting of details necessary to replicate studies and compare data across laboratories.

TEER output is a set of numerical values, which are used as an indicator of the integrity of a biological barrier. A decrease in TEER value below the limit of tissue integrity (if known), is interpreted as a compromised cellular barrier. TEER data has been used alone or in a weight of evidence alongside other data to evaluate cellular health or to assess toxicity in response to exposure to a test material (Wiese-Rischke et al., 2021). Correlating a change in TEER values in vitro to whether a biological effect in humans would be expected following exposure to a substance requires consideration of several parameters, including, but not limited to: (1) how much variability is observed in the normal range of TEER values for negative and positive controls (e.g., incubator and/or vehicle controls and relevant chemicals), and (2) how measured values in the test system compare to those previously reported for the same system (e.g., historical data for positive and negative controls).

Consideration of MIRTA recommendations will enable cross-laboratory comparisons, facilitate the incorporation of this assay into guideline studies, and facilitate the evaluation of study and data quality. Comparability and reproducibility of TEER assay data can also be strengthened by applying cause-and-effect analysis, which enables the identification of key sources of potential variability in a test method (Petersen et al. 2021), inclusion of sufficient details regarding study design (RIVER 2023; OECD 2018), and facilitated by FAIR (findable, accessible, interoperable, reusable) principles for improved data availability and reusability (Wilkinson et al. 2016).

As the RespTox Collaborative focuses on topics related to in vitro respiratory toxicity testing, the TEER reporting recommendations described in this paper apply to the respiratory toxicity field but can be adapted to other types of cellular barriers. This work was important to the RespTox Collaborative because inhalation is one of the primary routes through which exogenous substances may enter the body and scientific, legal, and ethical issues have led to a surge in the development of in vitro testing approaches (Movia et al. 2020). These in vitro tools, including the TEER assay, are being used to assess the effects of inhaled substances, making these MIRTA recommendations an important addition to this growing field.

## Supplementary information

Below is the link to the electronic supplementary material.

Supplementary file1 (DOCX 77 kb)
