# Supplementary material for: Minimum information for reporting on the TEER (trans-epithelial/endothelial electrical resistance) assay (MIRTA)
Source: Arch Toxicol. 2024 Oct 4;99(1):57–66. doi: 10.1007/s00204-024-03879-z (PMC11742365; doi:10.1007/s00204-024-03879-z)
Supplement: Supplementary file 1 — Supplementary file1 (DOCX 77 kb) [file 204_2024_3879_MOESM1_ESM.docx]

**Supplementary information**

**Minimum information for reporting on the TEER (trans-epithelial/endothelial electrical resistance) assay (MIRTA)**

Monita Sharma^1^, Erin Huber^2^, Emma Arnesdotter^3^, Holger P. Behrsing^4^, Adam Bettmann^5#^, David Brandwein^5$^, Samuel Constant^6^, Rahul Date^7^, Abhay Deshpande^7^, Eric Fabian^8^, Amit Gupta^9^, Robert Gutierrez^10^, Arno C. Gutleb^3^, Marie M. Hargrove^11^, Michael Hollings^12^, Victoria Hutter^13^, Annie M. Jarabek^14^, Yulia Kaluzhny^15*^, Robert Landsiedel^8,16^, Lawrence Milchak^5&^, Robert A. Moyer^9^, Jessica R. Murray^14^, Kathryn Page^17^, Manish Patel^7^, Stephanie N. Pearson^9^, Elijah J. Petersen^10^, Emily Reinke^18^, Nuria Roldan^1^, Clive Roper^19^, Jamie B. Scaglione^20^, Raja S Settivari^21^, Andreas O. Stucki^1^, Sandra Verstraelen^22^, Joanne L. Wallace^23^, Shaun McCullough ^2^, Amy J. Clippinger^1^

^1^PETA Science Consortium International e.V., 70499 Stuttgart, Germany

^2^Exposure and Protection, RTI International, 3040 East Cornwallis Road, Durham, NC, USA

^3^Luxemburg Institute of Science and Technology, Environmental Research and Innovation (ERIN) Department, 5 Avenue des Hauts-Fourneaux, 4362, Esch-sur-Alzette, Grand Duchy of Luxembourg

^4^Institute for In Vitro Sciences, Inc. Gaithersburg, MD 20878, USA

^5^3M Company, St. Paul, 55144, MN, USA

^6^Epithelix Sàrl, Chemin des Aulx 18, 1228 Plan-les-Ouates, Switzerland

^7^Jai Research Foundation, N. H. 48, Near Daman-Ganga bridge, Valvada 396105, Gujarat, India

^8^BASF SE, Experimental Toxicology and Ecology, 67056, Ludwigshafen, Germany

^9^Life Science Research, Battelle Memorial Institute, Columbus, Ohio 43201, USA

^10^Materials Measurement Laboratory, National Institute of Standards and Technology (NIST), Gaithersburg, MD 20899, USA

^11^Syngenta Crop Protection, 410 Swing Rd, Greensboro, North Carolina, 27409, USA.

^12^Labcorp Early Development Laboratories Ltd., North Yorkshire, HG3 1PY, UK

^13^ImmuONE Ltd, Science Building, College Lane, Hatfield, Herts AL10 9AB, UK; Centre for Topical Drug Delivery and Toxicology, University of Hertfordshire, College Lane Campus, Hatfield, Herts AL10 9AB, UK

^14^Center for Public Health and Environmental Assessment (CPHEA), Office of Research and Development, U.S. Environmental Protection Agency (EPA), Research Triangle Park, NC, 27711, USA

^15^MatTek Life Sciences, Ashland, Massachusetts 01721, USA

^16^Free University of Berlin, Pharmacy, Pharmacology and Toxicology, Berlin, Germany
^17^The Clorox Company, 4900 Johnson Dr, Pleasanton, CA 94588, United States.

^18^Inotiv, Morrisville, North Carolina, 27560, USA

^19^Roper Toxicology Consulting Limited, Edinburgh, EH3 6AD, UK,

^20^ScitoVation, LLC, Durham, NC 27709, USA

^21^Corteva Agriscience, Haskell R&D Center, Newark, DE, USA

^22^Flemish Institute for Technological Research (VITO), Environmental Intelligence Unit, 2400 Mol, Belgium

^23^Charles River Laboratories Edinburgh Ltd, Elphinstone Research Centre, Tranent, East Lothian EH33 2NE, UK

Current affiliation:

^#^PETA Science Consortium International e.V.

^$^Deceased

*InVitroTox Solutions Consulting

^&^Kimberly-Clark Corporation

Corresponding author: Monita Sharma, [monitas@thepsci.eu](mailto:monitas@thepsci.eu)

**A proposed template for reporting on the TEER assay in a publication**

Below is a proposed template for reporting on the TEER assay in a publication.

For this study, the **[type of voltohmmeter with manufacturer information]** was used with **[type of electrodes with manufacturer information, catalog number**]. The voltohmmeter was calibrated using the **[resistor]** and the electrodes were calibrated using **[electrolyte solution (type, concentration, and pH)]** and **[a calibration insert (type and catalogue number)]** at (**temperature)]**. The electrode was washed with **[disinfectant solution]** before use. For measurement, **[electrolyte solution (type and manufacturer)]** was equilibrated at **[temperature]** for **[time].** The **[test system (type, manufacturer, insert properties, if not otherwise reported)]** was removed from the incubator and the medium in the insert was **[temperature]** at the start of the experiment. A timer was set to record the duration for which the test system was out of the incubator. The test system **[was/wasn’t washed apically/basolaterally using (solution) for (time)**] and **[volume of electrolyte solution]** was added to the apical side and **[volume of electrolyte solution]** was added to the basolateral side. The electrodes were equilibrated in **[electrolyte solution]** for **[time]** before measurement. The electrodes were **[not rinsed/rinsed with (solution), and/or dried, and/or disinfected, etc.]** between measurements. After measurement, the electrolyte solution was removed via **[aspiration/pipetting]** and **[volume of culture medium]** was added to the **[basolateral side/apical side, if applicable]** of the insert before putting it back in the incubator. The inserts were outside the incubator for [**time**]. Resistance of the cells (R_cells_) was calculated by subtracting blank resistance (R_blank_) **[measured/default value]** from total measured resistance (R_total_).

R_cells_ = R_total_ – R_blank_

TEER was then calculated by multiplying R_cells_ by the cell culture insert surface area (S).

TEER (Ω·cm^2^) = R_cells_ (Ω) $\times$ S (cm^2^)

Raw values are reported in **[supplementary materials/repository, if applicable]**. Significant changes in TEER were assessed using **[statistical model].**
